# Isolated Perfused Hearts for Cardiovascular Research: An Old Dog with New Tricks

**DOI:** 10.1007/s12265-024-10517-7

**Published:** 2024-05-08

**Authors:** Tianshuo Yang, Zirui Liu, Songren Shu, Zhice Chen, Xiumeng Hua, Jiangping Song

**Affiliations:** 1 State Key Laboratory of Cardiovascular Disease, Fuwai Hospital, Chinese Academy of Medical Science, PUMC, 167 Beilishi Road, Xicheng District, Beijing, 100037 China; 2 Beijing Key Laboratory of Preclinical Research and Evaluation for Cardiovascular Implant Materials, Animal Experimental Centre, Fuwai Hospital, National Center for Cardiovascular Disease, Chinese Academy of Medical Sciences and Peking Union Medical College, Beijing, 100037 China; 3 Department of Cardiac Surgery, Fuwai Hospital, Chinese Academy of Medical Sciences and Peking Union Medical College, 167 Beilishi Road, Xicheng District, Beijing, 100037 China; 4 Shenzhen Key Laboratory of Cardiovascular Disease, Fuwai Hospital, Chinese Academy of Medical Sciences, Shenzhen, 518057 China

**Keywords:** Isolated heart model, Cardiac phenotyping, Ex vivo heart perfusion

## Abstract

In modern cardiovascular research, isolated perfused hearts have become cost-effective and highly reproducible tools to investigate the mechanisms of cardiovascular diseases (CVDs). Since they were first introduced in the nineteenth century, isolated perfused hearts have been extensively used for testing novel therapies, elucidating cardiac metabolic and electrophysiological activities, and modeling CVDs, including ischemic heart disease, arrhythmias, and hyperacute rejection. In recent years, ex vivo heart perfusion (EVHP) has shown potential in cardiac transplantation by allowing prolonged preservation and reconditioning of donor hearts. In this review, we summarize the evolution of the isolated perfused heart technique and its applications in cardiovascular research to help researchers comprehensively understand the capabilities of isolated heart models and provide guidance to use them to investigate various CVDs.

**Graphical Abstract:**

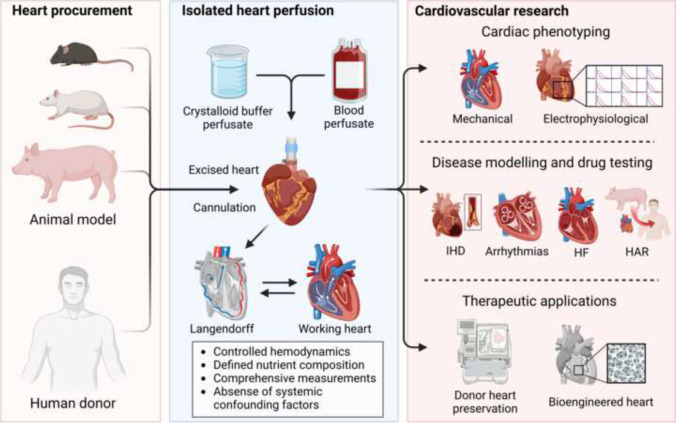

## Introduction

Cardiovascular diseases (CVDs) are the leading cause of mortality and disability worldwide, with approximately 18 million deaths attributed to CVDs each year [1, 2]. Despite significant advancements in cardiovascular medicine, substantial gaps remain in our therapeutic armament for CVDs [3]. Therefore, there is an urgent need to develop novel effective therapeutic approaches for CVDs.

Various animal disease models and cellular models have been applied to deepen our knowledge of CVDs [4]. Among these models, the isolated perfused heart technique stands out as a powerful experimental tool for exploring disease mechanisms and testing potential therapeutic strategies at the organ level (**Graphical abstract**). In this review, we will provide a brief introduction to the evolution of the isolated perfused heart technique and summarize the assessments for phenotyping isolated perfused hearts, with a focus on the recent applications of isolated perfused hearts in disease modeling to investigate disease mechanisms and develop novel therapies for CVDs.

## Evolution of Isolated Heart Perfusion Technique

**Fig. 1:**
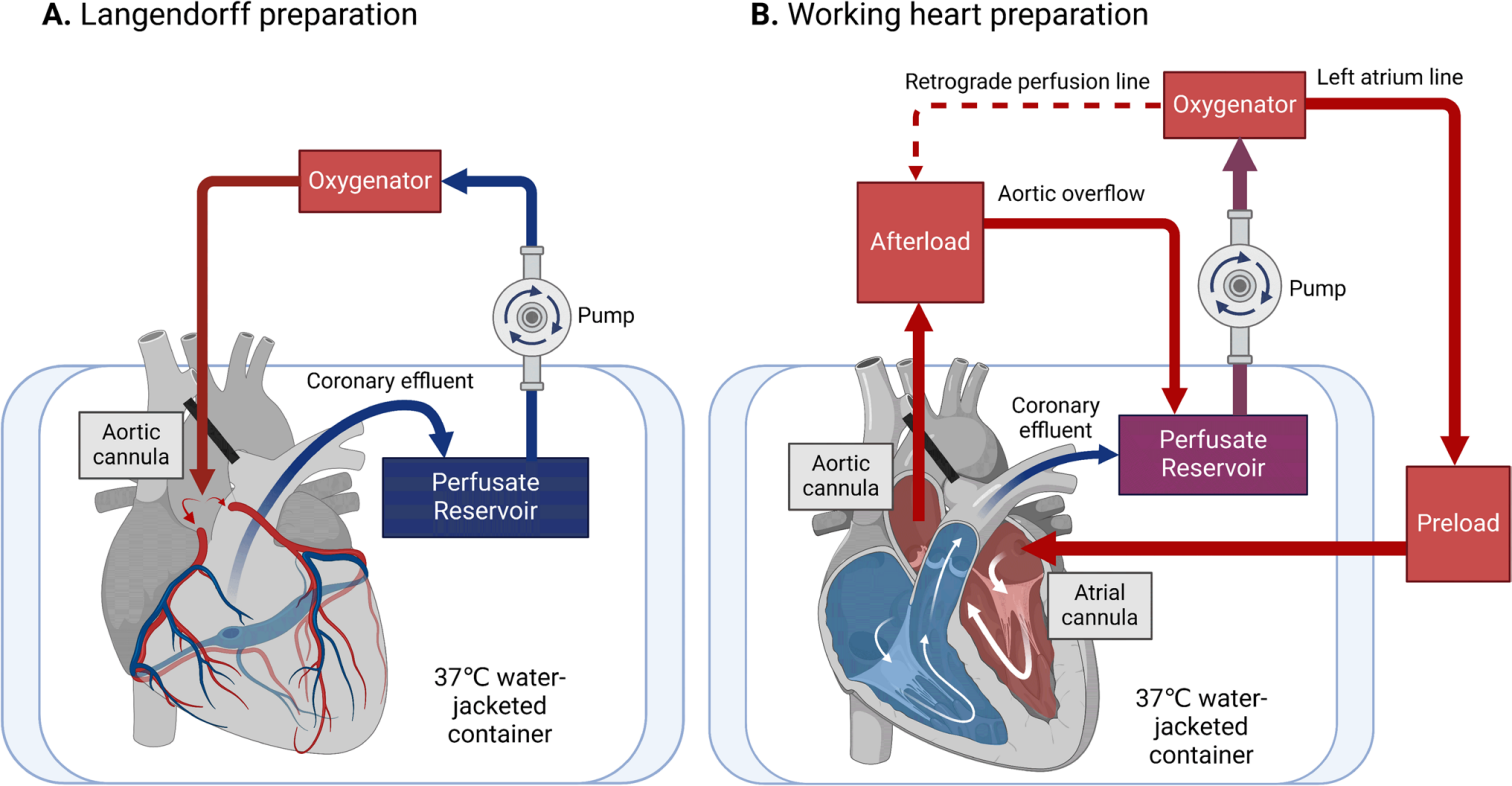
Schematic diagram of isolated heart preparations. In the Langendorff preparation (**A**), the excised heart is cannulated through the aorta. Oxygenated perfusate flows retrogradely down the aorta and enters the coronary circulation. After perfusing the whole heart, the perfusate is drained via the pulmonary artery and collected in a reservoir for re-oxygenation. In working heart preparation (**B**), an extra cannula is inserted into the left atrium to provide preload. The left ventricle ejects, supports aortic pressure and maintains coronary perfusion

The idea of studying cardiac physiology in isolated perfused hearts was first developed and realized by Carl Ludwig and his students in the middle of the nineteenth century [5]. However, they failed to resuscitate mammalian hearts after being excised from the animal. In the late nineteenth century, German physiologist Oskar Langendorff overcame the hurdle of perfusing isolated mammalian hearts by applying a retrograde heart perfusion technique [5, 6]. Briefly, the excised heart is cannulated into the aorta, with blood flowing down the aorta, opposite of the physiological flow. The aortic valve is closed because of hydrostatic perfusion pressure, and the blood then flows into the coronary arteries to perfuse the whole heart. The venous blood is drained via the coronary sinus into the right atria, finally ejected through the pulmonary artery, and collected in a reservoir. This retrograde-perfused heart method according to Langendorff (known as “Langendorff heart”) has become the basis of all modern isolated heart preparations. A schematic diagram of the modern Langendorff heart preparation is shown in Fig. 1A.

In 1967, two American physiologists, Neely and Morgan, first described an isolated “working heart” preparation to mimic the physiological workload of the heart [7]. With an extra cannula inserted in the left atria, the isolated heart is switched to “working mode” to eject perfusate with a controlled preload pressure, comparable to the hearts under physiological conditions (Fig. 1B). This preparation allows functional and metabolic assessments of hearts in variable preload and afterload [8].

## Cardiac Phenotyping with Isolated Perfused Hearts

**Fig. 2:**
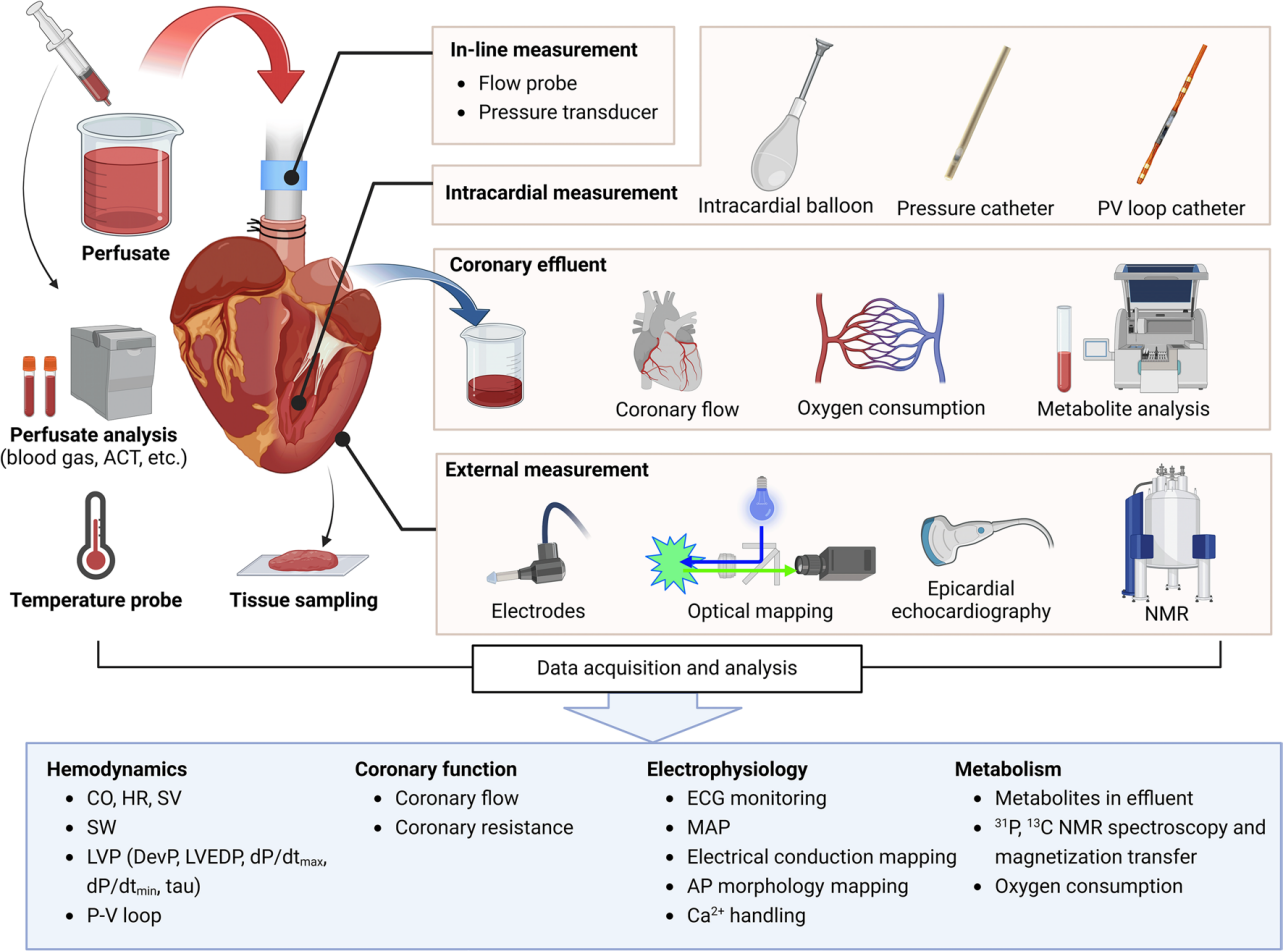
Cardiac phenotyping with isolated perfused hearts. During isolated heart perfusion, monitoring data can be obtained with in-line, intracardial and epicardial probes. including hemodynamics, coronary function, electrophysiology, and metabolic function. ACT, activated clotting time; LV, left ventricular; PV loop, pressure–volume loop; CO, cardiac output; HR, heart rate; SV, stroke volume; SW, stroke work; LVP, left ventricular pressure; DevP, developed pressure; LVEDP, left ventricular end-diastolic pressure; dP/dtmax, the maximal derivative of left ventricular pressure; dP/dtmin, the minimal derivative of left ventricular pressure; MAP, monophasic action potential; AP, action potential; NMR, nuclear magnetic resonance

Isolated perfused hearts provide a unique platform for multidimensional cardiac phenotyping with high reproducibility in a rapid and cost-effective manner. In addition to conventional monitoring indices such as ECG and temperature, more detailed measures for cardiac function, electrical activities and metabolic activities can be performed in isolated perfused hearts (Fig. 2).

### Evaluation of Mechanical Cardiac Function

In non-ejecting Langendorff hearts, cardiac function can be evaluated by inserting a balloon in the left ventricle, which is attached to a pressure transducer. Left ventricular pressure (LVP) in isovolumetric contracting Langendorff hearts can be monitored. In working heart preparations, cardiac function can be evaluated by hemodynamic parameters such as cardiac output (CO) and stroke work (SW) using in-line aortic pressure and flow probes. Moreover, the ventricular function of the physiological ejecting hearts can be evaluated more accurately by inserting a pressure catheter into the left ventricle to monitor LVP.

The pressure–volume (PV) loop catheter allows for the simultaneous acquisition of left ventricular volume (LVV) and LVP data, making it the gold standard measure for ventricular function. PV loop monitoring also enables the calculation of functional measures, including stroke volume (SV), end-systolic pressure–volume relationship (ESPVR), end-diastolic pressure–volume relationship (EDPVR), and the relaxation time constant, Tau (τ). Epicardial echocardiography can be performed during heart perfusion to gain information on the diameters of the heart chambers, ventricular wall thickness and valve function [9].

### Electrophysiological Assessment

Electrocardiogram (ECG) can be monitored easily on the perfused heart. Meanwhile, more detailed electric activities can be recorded with electrode- and optical-based methods.

Monophasic action potentials (MAPs) of the myocardium can be measured by pressing electrodes into the cardiac tissue [10]. MAPs provide valuable insights into the repolarization capability of local myocardial tissue. The development of optical mapping technology revolutionized the pattern of cardiac electrophysiology research. Cardiac optical mapping techniques leverage high-precision optical imaging systems in combination with fluorescent dyes to achieve high spatiotemporal resolution in monitoring membrane potentials and calcium transients [11, 12]. Langendorff perfused hearts allow the infusion of excitation–contraction uncoupler blebbistatin to reduce motion artifacts during cardiac optical mapping. After dye loading, fluorescence excited by photons allows for the detection and recording of fluorescence intensity changes on the surface of the heart through the imaging system. Various indices, such as action potential activation map, action potential duration (APD), and alternans, can be calculated and analyzed to investigate cardiac arrhythmia mechanisms [13, 14] or characterize the action potentials of the heart [15, 16]. In addition, the isolated perfused heart technique enables the application of programmed stimulation protocols to test arrhythmia vulnerability, without concern about causing hemodynamic instability as in in vivo models [17].

### Metabolic Assessment

The isolated perfused heart offers the advantage of complete control of substrates, hormones, and oxygen without confounding factors in vivo. When perfused with metabolic substrates labeled by isotopes, the concentration of metabolites and metabolic flux can be measured directly in combination with radiotracer analysis, nuclear magnetic resonance (NMR), mass spectrometry (MS), and other approaches.

However, the process of ex vivo perfusion can alter the metabolic activity of the heart, such as phosphorylation level of kinases and perfusion-induced oxidative stress [18, 19]. The assessment and application of isolated perfused hearts in cardiac metabolic research have been comprehensively reviewed elsewhere [8, 20].

## Isolated Perfused Hearts for Disease Modeling and Drug Testing

Isolated perfused hearts provide a controlled environment to mimic cardiac diseases and evaluate therapeutic interventions. By recapitulating disease conditions and introducing potential treatments, this technique offers valuable insights into disease mechanisms and accelerates the development of novel therapeutic approaches.

### Ischemic Heart Disease

**Fig. 3:**
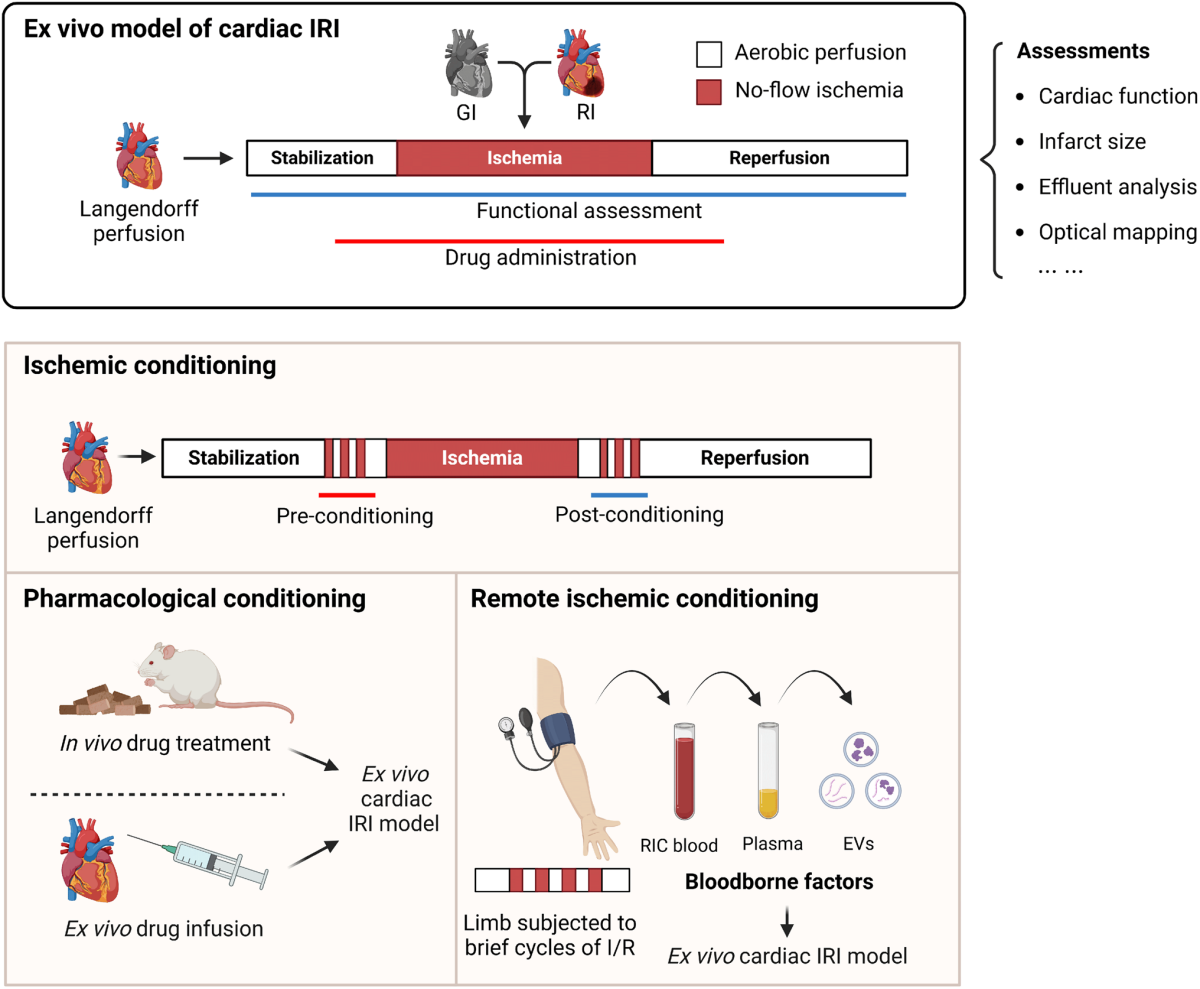
Ex vivo model of cardiac ischemia and reperfusion injury. In isolated perfused hearts, ischemia can be easily induced either by stopping the perfusion flow or transient ligation of the LAD. Functional and electrophysiological indices can be measured throughout the experiment. Cardioprotective agents or pathway inhibitors can be administered chronically in vivo or acutely infused ex vivo before, during and after ischemia. At the end of the experiment, infarct size, effluent component analysis, protein analysis and other assays can be performed. IRI, ischemia and reperfusion injury; GI, global ischemia; RI, regional ischemia; I/R, ischemia/reperfusion; EV extracellular vehicle

The ex vivo model of cardiac ischemic-reperfusion injury (IRI) is well established and has been extensively used in cardiac research for decades [21]. In Langendorff hearts, ischemia and reperfusion can be induced by transient ligation of the left anterior descending coronary artery for regional ischemia or simply stopping the perfusion inflow for global ischemia. After a set episode of ischemia, the oxygenated perfusion flow can be resumed for a period of time to mimic reperfusion. Cardiac function, infarct size, cell death and other assessments can be performed to evaluate IRI in the heart (Fig. 3).

Ex vivo cardiac IRI models have been extensively used to discover novel cardioprotective agents or validate the cardioprotective effects of existing drugs, either by chronic administration in vivo or acute infusion ex vivo [22–25].

Remote ischemic conditioning (RIC) by brief cycles of ischemia–reperfusion in a remote organ (limb) has been shown to effectively attenuate myocardial IRI in various preclinical models and in patients with acute cardiac IRI injury [26–29]. The exact underlying mechanism of RIC remains elusive. Neuronal and humoral factors are involved in the signal transfer process, such as the activation of peripheral sensory fibers and the release of nitric oxide and microRNAs [26]. To investigate the kinetics of humoral factors of RIC, Hildebrandt et al. collected plasma from volunteers at different time points after RIC [30]. Dialysates of plasma samples were infused in isolated mouse hearts and subjected to global ischemia and reperfusion. As a result, dialysates obtained 5 min to 6 days after RIC significantly reduced infarct size and increased STAT3 phosphorylation compared to baseline dialysate. In another study, extracellular vehicles (EVs) separated from the plasma of human volunteers after RIC reduced infarct size in an ex vivo IRI model of rat hearts, and researchers identified miR-16-5p, miR-144-3p and miR-451a as significantly upregulated in RIC EVs as potential effectors of the RIC modulation effect [31].

### Cardiac Electrophysiology and Arrhythmia

Traditional application of isolated perfused hearts to study arrhythmia includes testing the anti- and pro-arrhythmic effects of drugs [32]. In the past 10 years, the development of optical electrophysiology and new materials has significantly deepened our understanding of cardiac electrophysiology and arrhythmia, such as atrial fibrillation (AF) and ventricular tachycardia (VT).

AF is the most common sustained arrhythmia and the most important risk factor for stroke [33]. For a long time in history, AF has been deemed aperiodic or random. Until the twentieth century, with the development of the optical mapping technique, spatiotemporal periodicity was observed during an induced AF model in isolated perfused hearts [34, 35]. These studies provided evidence for the hypothesis that stable, self-sustained rotors exist in the atria and that high-frequency activation by such rotors results in complex patterns of activation that characterize AF [13, 36]. However, current therapy remains limited, partly due to the inability of single-surface electrode mapping to localize AF drivers in the complex human heart. Studies in ex vivo perfused human hearts have provided deeper insights into AF mechanisms to guide future treatment. For example, Hansen et al. utilized high-resolution transmural optical mapping approaches coupled with 3D contrast-enhanced MRI to define the precise functional and structural features of 3D microanatomic reentrant AF drivers [37–39].

VT, including ventricular fibrillation (VF), is the most common cause of sudden cardiac death [40]. In isolated mammalian hearts, rotors were also observed to contribute to the mechanism of VF [41, 42]. This was confirmed in Langendorff-perfused human hearts, in which rotors and even scroll waves were observed with electrical and transmural optical mapping during early VF [43]. Gene mutations are one of the major causes of VF. In isolated perfused hearts of mice with RyR2/RyR2^R4496C^ or KCNJ2-R67Q transgene, increased focal activity and reentry and more VT occurrence were observed with optical mapping after epinephrine and caffeine induction [44, 45]. In Langendorff-perfused hearts, Moreno et al. found that the effects of acute mechanical load and ischemia on VF frequency and dynamics attenuate in heart failure compared to normal hearts [46].

Another major advancement in cardiac electrophysiology is the application of optogenetics, which enables the control of selected cells after being modified to express light-sensitive proteins [47]. The emergence of cardiac optogenetics allows for the development of all-optical electrophysiology, allowing precise light-induced actuation of hearts with high spatiotemporal resolution imaging of morphology and conduction patterns of action potentials and calcium transients [48, 49]. Recent proof-of-concept studies have shown successful VT termination in isolated rodent hearts [50–52].

The isolated perfused hearts have enabled preliminary testing of novel tissue-compatible biointerfaces for long-term monitoring and even treatment of arrhythmias. In recent years, various implantable devices, such as stretchable and adhesive epicardial membranes, have been introduced and tested in isolated animal or human hearts (Table 1). In these studies, the interaction of the devices and the isolated beating hearts were analyzed to demonstrate the superiority of this device and the feasibility of in vivo studies and clinical translation.

**Table 1:** Recent studies of electric biointerface tested with ex vivo heart models

Study	Ex vivo model	Usage	Sensors	Therapeutic functions
Fang, 2017 [93]	Rabbit	Epicardial membrane	Electrode array	-
Liu, 2020 [94]	Pig, rabbit	Epicardial membrane	Electrode array	-
Chen, 2021 [95]	Mouse, rat	Transparent epicardial membrane	Electrode array	-
Li, 2023 [96]	Rat	Bioadhensive epicardial membrane	Organic electrochemical transistors	-
Li, 2023 [97]	Rat	Ultrasoft device with a soft interlayer	Electrode array	
Prominski, 2022 [98]	Rat	Epicardial membrane	Electrode array	Optical stimulation
Han, 2020 [99]	Rabbit, human	Intraventricular catheter	Electrode array, temperature sensor array, pressure sensor	RFA, IRE
Xu, 2014 [100]	Rabbit	3D printed epicardial membrane	ECG sensor, Si strain gauge, pH sensor, temperature sensor	Electrical, thermal and optical stimulation
Lin, 2023 [101]	Mouse	Graphene Cardiac Tattoo	Electrode array	Pacing

*RFA* radiofrequency ablation, *IRE* irreversible electroporation

### Cardiac Allotransplantation

After heart transplantation, 10% to 20% of the donor grafts develop primary graft dysfunction (PGD), causing 39% of the mortality after 1 month of transplantation [53, 54]. Ex vivo perfused hearts provide a useful tool to explore the value of novel treatments to prevent PGD. In a recent study, researchers showed the protective effect of the histone deacetylase inhibitor valproic acid (VPA) in static cold storage (SCS)-preserved hearts [55]. The contractility and diastolic ability of mouse hearts evaluated by dP/dt_max_ and dP/dt_min_ were assessed to evaluate the improvement of cardiac preservation quality by administration of VPA and its downstream effector 4-octyl itaconate. In addition, porcine hearts preserved with VPA showed significantly better function in ex vivo working heart preparation compared to controls, exhibiting translational potential in clinical settings [55].

### Pig-to-Human Cardiac Xenotransplantation

Pig-to-human cardiac xenotransplantation is a promising solution to bridge the gap of insufficient heart donors [56]. Transplanting a porcine heart into the human body can result in a strong rejection reaction within minutes to hours, causing dysfunction of the graft, known as hyperacute rejection (HAR). Ex vivo xeno-perfusion models are widely used to investigate the HAR mechanism. Forty et al. first utilized an isolated rabbit heart model in the 1990s to investigate the mechanism of xenograft HAR [57–59]. In the following years, with clinically relevant xeno-perfusion models that perfused pig hearts with human blood, studies identified blood components, including xeno-antibodies [60–64], the complement system [65–67], the coagulation system [61, 68], endothelial cells [69], and ABO blood group [67], that play important roles in the pathogenesis of HAR.

Ex vivo heart models were also used to evaluate the effect of gene modification strategies on HAR prevention. For example, perfusion of human complement regulatory protein hDAF, hCD46, or hCD59 transgenic pig hearts with human blood showed significantly prolonged survival compared to control [70–72]. In the past decade, a large number of novel gene modifications have been proposed for clinical xenotransplantation [73]. Abicht et al. utilized a xeno-perfusion model to validate the protective role of hemoxygenase-1/A20 and HLA-E/β2m transgenic pig hearts [74, 75] before being tested in non-human primate models. The studies using pig-to-human cardiac xeno-perfusion models are summarized in Table 2.

**Table 2:** Xenotransplantation studies using clinically related xeno-perfusion heart models

Intervention	Perfusion Mode	Outcome	Reference
None	LD	Loss of function within 15–30 min. IgM, IgG and complement deposition	Kirk, 1993 [61]
	WH	Demonstrated the central role of complement in the destruction of pig-to-man xenografts	Dunning, 1994 [62]
	BiWH	Cytotoxic T cells were activated within 3 h, accompanied by upregulation of anti-inflammatory microRNAs	Tomasi, 2021 [102]
Complement System			
Heat-treated	WH	Increased survival and stroke work	Dunning, 1994 [62]
CVF	WH	Increased survival and stroke work, no C3 deposition observed	Dunning, 1994 [62]
sCR1	LD	Marked prolonged survival, cardiac function preserved, no C3i/C3b deposition observed	Pruitt, 1994 [60]
Anti-C5 monoclonal antibody	LD	Prolonged survival, inhibited complement activation pathway	Kroshus, 1995 [63]
Soluble hCD46 and hCD55	LD	Prolonged survival	Kroshus, 2000[64]
C3 inhibitor Cp40	BiWH	Inhibited C3 activation, reduced cell death, preserved cardiac function	Abicht, 2017 [103]
Coagulation System			
GP IIb/IIIa inhibitor	WH	Prolonged survival, preserved cardiac function	Brandl, 2007 [104]
hirudin	WH	Prolonged survival, preserved cardiac function, reduced tissue injury, thrombosis and complement deposition	Brandl, 2007 [104]
Xenoantibody Depletion			
IgM and IgG depletion	LD	Anti-pig antibodies markedly reduced, prolonged survival, preserved cardiac function	Kroshus, 1995 [65]
IgM depletion	LD	Markedly reduced IgM and complement deposition, prolonged survival, activity of complement system inhibited	Kroshus, 1996 [66]
Ischemic Time	WH	Prolonged cold storage time showed controversial beneficial effects in xeno-perfused heart model	Brenner, 2000 [105]
Genetic modification			
hDAF-tg	LD	Attenuated HAR, preserved cardiac function and metabolism	Smolenski, 2007 [106]
	WH	Increased survival and stroke work, decreased myocardial injury and edema	Schmoeckel, 1996 [70]
hCD59-tg	LD	Prolonged survival, markedly decreased C9 deposition	Kroshus, 1996 [72]
HO-1/hCD46/hCD55/hCD59	BiWH	Preserved cardiac function and coronary perfusion, reduced myocardial edema and injury	Abicht, 2016 [74]
GTKO/hCD46/HLA-E/hβ2-mg	BiWH	Reduced IgG deposition, NK cell infiltration, inhibited complement system activation, preserved cardiac function	Abicht, 2018 [75]

*LD* Langendorff mode, *WH* working heart mode, *BiWH* bi-ventricular working heart mode, *CVF* cobra venom factor, *sCR1* soluble complement receptor 1, *MAb* monoclonal antibody, *hDAF* human decay accelerating factor, *tg* transgene, *GTKO* alpha-1,3-galactosyltransferase knockout, *HLA* human leukocyte antigen, hβ2-mg, human β2microglobulin

## Donor Heart Preservation and Reconditioning

The isolated perfused heart technique provides an opportunity to deliver interventions to the heart during perfusion to optimize the heart for transplantation. Ex vivo heart perfusion (EVHP) provides a means of constantly supplying oxygen and nutrients to beating donor hearts while removing metabolic waste products from the myocardium. EVHP greatly reduced ischemic time compared to static cold storage (SCS), minimizing the ischemia–reperfusion injury of the donor heart during transportation [76].

A recent randomized controlled trial showed higher survival of patients at 6 months and 1 year with EVHP preserved DCD hearts than with SCS-preserved hearts [77]. EVHP also provides an opportunity to evaluate the function of donor hearts. However, due to the non-physiological workload, the evaluation of mechanical function and the metabolic marker lactate in EVHP hearts is suboptimal [78, 79]. Assessing cardiac function in working heart mode has been achieved by EVHP devices using pre-clinical models [80–82]. In non-beating hyperthermic perfused hearts, microvascular flow has been proposed for predicting the mechanical function of the graft [83].

**Fig. 4:**
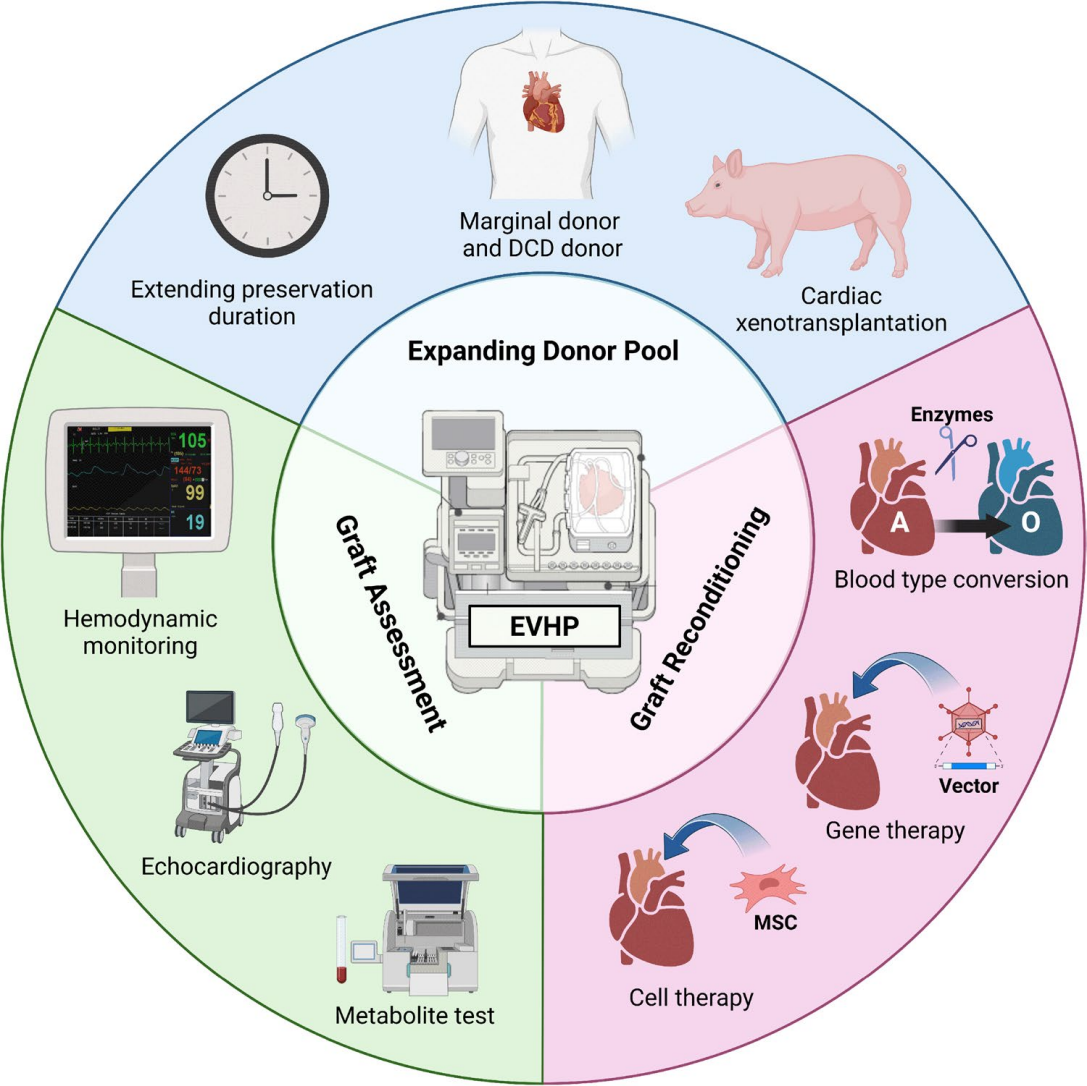
EVHP applications in clinical cardiac transplantation. EVHP has been utilized to improve donor heart preservation quality. It also expands the donor pool by enabling the usage of marginal, DCD, and even xenogeneic donors. While perfused ex vivo, multiparametric assessments and functional monitoring can be performed to evaluate donor heart viability before transplant. EVHP also provides a platform to deliver therapies for donor heart reconditioning, such as cell therapy, gene therapy and other treatments to alleviate immunological injury to the graft. EVHP, ex vivo heart perfusion; DCD, donation after circulatory death

EVHP is a perfect platform to deliver therapy to the heart at high concentrations without being affected by other organs of the body. For example, ex vivo deliver of transfection reagents containing siRNAs to the donor heart improved cardiac tissue survival and function in isolated working heart and orthotopic heart transplant models [84]. Mesenchymal stem cell (MSC) transplantation is another promising approach to improve the quality of margin donor hearts [85–87]. Gene transfer using adeno-associated viral vectors (AAVs) in OCS has been proven feasible by several studies [88–90], showing great potential for the upregulation of co-inhibitory molecules to protect the graft from immunological injury or deliver gene therapy in the hearts of donors with mutations. Future directions for reconditioning donor organs in EVHP include nanoparticles [91] and enzymatic conversion of discordant antigens (Fig. 4) [92].

## Summary and Perspectives

The isolated perfused heart technique is a time-tested experimental method with versatile applications in cardiovascular research. It allows comprehensive evaluation of cardiac function, electrophysiology, and metabolism without systematic effects. Crucially, it enables disease modeling for conditions like ischemic heart disease and arrhythmia, aiding in the exploration of novel therapeutic approaches. Moreover, it plays an increasingly important role in cardiac transplantation, facilitating studies on graft preservation and xenotransplantation. With ongoing advancements, such as optogenetics and gene therapy, this technique continues to promise deeper insights into cardiovascular pathophysiology and potential treatments, offering hope for improved patient outcomes.

## Abbreviations

*AAV*: Adeno-associated viral vector
*AF *: Atrial fibrillation
*CVD*: Cardiovascular disease
*DCD *: Donation after circulatory death
*EV*: Extracellular vehicle
*EVHP*: Ex vivo heart perfusion
*HAR*: Hyperacute rejection
*HF*: Heart failure
*IRI*: Ischemic-reperfusion injury
*MAP*: Monophasic action potential
*MS*: Mass spectrometry
*MSC*: Mesenchymal stem cell
*NMR*: nuclear magnetic resonance
*PGD*: primary graft dysfunction
*RIC*: remote ischemic conditioning
*SCS*: static cold storage
*VF*: ventricular fibrillation
*VPA*: valproic acid
*VT*: ventricular tachycardia
